# Supplementary material for: Exercise therapy to improve cervical proprioception in individuals with asymptomatic forward head posture: A systematic review of randomized controlled trials
Source: PLoS One. 2025 Sep 18;20(9):e0330665. doi: 10.1371/journal.pone.0330665 (PMC12445534; doi:10.1371/journal.pone.0330665)
Supplement: Attachment — Submitted filename: PONE-D-25-29149_reviewer_AO.pdf [file pone.0330665.s002.pdf]

PLOS ONE

Exercise therapy for cervical proprioception in individuals with asymptomatic forward head posture: a systematic review

--Manuscript Draft--

**Manuscript Number:**	PONE-D-25-29149
**Article Type:**	Research Article
**Full Title:**	Exercise therapy for cervical proprioception in individuals with asymptomatic forward head posture: a systematic review
**Short Title:**	Exercise for Cervical Proprioception
**Corresponding Author:**	Mohammad Alimoradi<br>Shahid Bahonar University of Kerman<br>Kerman, --- Select One --- IRAN, ISLAMIC REPUBLIC OF
**Keywords:**	proprioception; Posture; cervical vertebrae; exercise therapy
**Abstract:**	Background<br><br>Forward head posture (FHP) is a common musculoskeletal condition associated with impaired cervical proprioception, which compromises postural control and neuromuscular function. Exercise-based interventions have been proposed to address proprioceptive deficits in FHP, but their effectiveness remains unclear. This systematic review aimed to evaluate the impact of exercise-based interventions on cervical proprioception in individuals with FHP.<br><br>Methods<br><br>A systematic search of PubMed, Scopus, and Web of Science was conducted up to March 28, 2025. Randomized controlled trials (RCTs) assessing exercise interventions on cervical proprioception in FHP were included. Risk of bias was assessed using the RoB-2 tool. Due to heterogeneity in outcome measures, a narrative synthesis was conducted, supported by effect size (ES) calculations.<br><br>Results<br><br>Nine RCTs involving 367 participants were included. Interventions ranged from cervical stabilization exercises (most common) to whole-body vibration, backward walking, and muscle energy techniques. ESs varied from trivial to nearly perfect, with trivial-to-very-large improvements observed in joint position sense and joint position error. Cervical stabilization exercises demonstrated the most consistent benefits, particularly in rotation and flexion tasks. Risk of bias was generally rated as "some concerns" due to lack of blinding and variability in outcome measures.<br><br>Conclusion<br><br>Exercise-based interventions, especially cervical stabilization exercises, can significantly improve cervical proprioception in individuals with FHP. However, methodological heterogeneity and diagnostic inconsistencies limit definitive conclusions. Future studies should standardize diagnostic criteria, outcome assessments, and investigate long-term effects across diverse populations.
**Order of Authors:**	Ali Asghar Norasteh
	Kimia Karimi
	Shabnam Faraji
	Fereshteh Ejlali
	Mohammad Alghosi
	Mohammad Alimoradi

Powered by Editorial Manager® and ProduXion Manager® from Aries Systems Corporation

	Hamed Zarei
**Additional Information:**	
**Question**	**Response**
**Financial Disclosure**<br><br>Enter a financial disclosure statement that describes the sources of funding for the work included in this submission. Review the submission guidelines for detailed requirements. View published research articles from *PLOS ONE* for specific examples.<br><br>This statement is required for submission and **will appear in the published article** if the submission is accepted. Please make sure it is accurate.<br><br>**Funded studies**<br>Enter a statement with the following details:<br>• Initials of the authors who received each award<br>• Grant numbers awarded to each author<br>• The full name of each funder<br>• URL of each funder website<br>• Did the sponsors or funders play any role in the study design, data collection and analysis, decision to publish, or preparation of the manuscript?<br><br>Did you receive funding for this work?	The author(s) received no specific funding for this work.
**Competing Interests**<br><br>Use the instructions below to enter a competing interest statement for this submission. On behalf of all authors, disclose any competing interests that could be perceived to bias this work—acknowledging all financial support and any other relevant financial or non-financial competing interests.<br><br>This statement is required for submission and **will appear in the published article** if the submission is accepted. Please make sure it is accurate and that any funding	The authors have declared that no competing interests exist.

sources listed in your Funding Information later in the submission form are also declared in your Financial Disclosure statement.<br><br>View published research articles from *PLOS ONE* for specific examples.<br><br>**NO authors have competing interests**<br><br>Enter: *The authors have declared that no competing interests exist.*<br><br>**Authors with competing interests**<br><br>Enter competing interest details beginning with this statement:<br><br>*I have read the journal's policy and the authors of this manuscript have the following competing interests: [insert competing interests here]*<br><br>* typeset	
**Ethics Statement**<br><br>Enter an ethics statement for this submission. This statement is required if the study involved:<br><br>• Human participants<br>• Human specimens or tissue<br>• Vertebrate animals or cephalopods<br>• Vertebrate embryos or tissues<br>• Field research<br><br>Write "N/A" if the submission does not require an ethics statement.<br><br>General guidance is provided below. Consult the submission guidelines for detailed instructions. **Make sure that all information entered here is included in the Methods section of the manuscript.**	N/A

**Format for specific study types**<br><br>**Human Subject Research (involving human participants and/or tissue)**<br>• Give the name of the institutional review board or ethics committee that approved the study<br>• Include the approval number and/or a statement indicating approval of this research<br>• Indicate the form of consent obtained (written/oral) or the reason that consent was not obtained (e.g. the data were analyzed anonymously)<br><br>**Animal Research (involving vertebrate animals, embryos or tissues)**<br>• Provide the name of the Institutional Animal Care and Use Committee (IACUC) or other relevant ethics board that reviewed the study protocol, and indicate whether they approved this research or granted a formal waiver of ethical approval<br>• Include an approval number if one was obtained<br>• If the study involved *non-human primates*, add *additional details* about animal welfare and steps taken to ameliorate suffering<br>• If anesthesia, euthanasia, or any kind of animal sacrifice is part of the study, include briefly which substances and/or methods were applied<br><br>**Field Research**<br><br>Include the following details if this study involves the collection of plant, animal, or other materials from a natural setting:<br>• Field permit number<br>• Name of the institution or relevant body that granted permission	
**Data Availability**<br><br>Authors are required to make all data underlying the findings described fully available, without restriction, and from the time of publication. PLOS allows rare exceptions to address legal and ethical concerns. See the PLOS Data Policy and FAQ for detailed information.	Yes - all data are fully available without restriction

A Data Availability Statement describing where the data can be found is required at submission. Your answers to this question constitute the Data Availability Statement and **will be published in the article**, if accepted.<br><br>**Important:** Stating 'data available on request from the author' is not sufficient. If your data are only available upon request, select 'No' for the first question and explain your exceptional situation in the text box.<br><br>Do the authors confirm that all data underlying the findings described in their manuscript are fully available without restriction?	
**Describe where the data may be found in full sentences. If you are copying our sample text, replace any instances of XXX with the appropriate details.**<br><br>• If the data are **held or will be held in a public repository**, include URLs, accession numbers or DOIs. If this information will only be available after acceptance, indicate this by ticking the box below. For example: *All XXX files are available from the XXX database (accession number(s) XXX, XXX.)*.<br>• If the data are all contained **within the manuscript and/or Supporting Information files**, enter the following: *All relevant data are within the manuscript and its Supporting Information files.*<br>• If neither of these applies but you are able to provide **details of access elsewhere**, with or without limitations, please do so. For example:<br><br>*Data cannot be shared publicly because of [XXX]. Data are available from the XXX Institutional Data Access / Ethics Committee (contact via XXX) for researchers who meet the criteria for access to confidential data.*<br><br>*The data underlying the results presented in the study are available from (include the name of the third party*	All relevant data are within the manuscript and its Supporting Information files.

and contact information or URL).<br>• This text is appropriate if the data are owned by a third party and authors do not have permission to share the data.<br><br>* typeset	
Additional data availability information:	

Manuscript

Click here to access/download;Manuscript;Manuscript.docx

Exercise therapy for cervical proprioception in individuals with asymptomatic forward head posture: a systematic review

Running Title: Exercise for Cervical Proprioception

Ali Asghar Norasteh[1], Kimia Karimi[2], Shabnam Faraji[3], Fereshteh Ejlali[4], Mohammad Alghosi[2, 5], Mohammad Alimoradi[6, *], Hamed Zarei[2]

1. Department of Physiotherapy, Faculty of Medicine, Guilan University of Medical Sciences, Rasht, Iran. Email: Asgharnorasteh@yahoo.com
2. Department of Sports Injury and Corrective Exercise, Faculty of Physical Education & Sport Sciences, University of Guilan, Rasht, Iran. Email: Kimia.Karimi.ce@gmail.com; Mohammadalghosi@phd.guilan.ac.ir; Zareei.h@yahoo.com
3. Department of Sport Injuries and Biomechanics, Faculty of Sport Sciences and Health, University of Tehran, Tehran, Iran. faraji.shabnam@ut.ac.ir
4. Department of Physical Education and Exercise Science, Islamic Azad University, Central Tehran Branch (Velayat Campus), Tehran, Iran. Fereshteh16ejlali@gmail.com
5. Department of Physical Education and Sport Science, National University of Skill (NUS), Tehran, Iran.
6. Department of Sports Injuries and Corrective Exercises, Faculty of Sports Sciences, Shahid Bahonar University of Kerman, Kerman, Iran.

[*]**Corresponding author:** Mohammad Alimoradi
Tel: +98-991-384-3926; Email: Malimoradi@sport.uk.ac.ir

Registration: International Prospective Register of Systematic Reviews CRD42023488327

Acknowledgments: The authors sincerely thank the peer reviewers for their valuable feedback and support in improving the manuscript.

Funding: This research did not receive any specific grant from funding agencies in the public, commercial, or not-for-profit sectors.

Conflict of Interest: The authors declare that they have no conflicts of interest relevant to the content of this review.

Author Contributions

All authors meet the ICMJE criteria for authorship and have contributed substantially to the work. Specifically:

- **Ali Asghar Norasteh** and **Kimia Karimi** contributed to the conception and design of the review, performed initial data screening, and drafted the original manuscript.
- **Shabnam Faraji**, **Fereshteh Ejlali**, **Mohammad Alghosi**, **Mohammad Alimoradi**, and **Hamed Zarei** contributed to study selection, data extraction, risk of bias assessment, and interpretation of findings.

- **Mohammad Alimoradi** supervised the review process, provided critical revisions for intellectual content, and is the corresponding author responsible for the integrity of the work as a whole.

All authors reviewed and approved the final manuscript and agree to be accountable for all aspects of the work.

Abstract

Background:

Forward head posture (FHP) is a common musculoskeletal condition associated with impaired cervical proprioception, which compromises postural control and neuromuscular function. Exercise-based interventions have been proposed to address proprioceptive deficits in FHP, but their effectiveness remains unclear. This systematic review aimed to evaluate the impact of exercise-based interventions on cervical proprioception in individuals with FHP.

Methods:

A systematic search of PubMed, Scopus, and Web of Science was conducted up to March 28, 2025. Randomized controlled trials (RCTs) assessing exercise interventions on cervical proprioception in FHP were included. Risk of bias was assessed using the RoB-2 tool. Due to heterogeneity in outcome measures, a narrative synthesis was conducted, supported by effect size (ES) calculations.

Results:

Nine RCTs involving 367 participants were included. Interventions ranged from cervical stabilization exercises (most common) to whole-body vibration, backward walking, and muscle energy techniques. ESs varied from trivial to nearly perfect, with trivial-to-very-large improvements observed in joint position sense and joint position error. Cervical stabilization exercises demonstrated the most consistent benefits, particularly in rotation and flexion tasks. Risk of bias was generally rated as "some concerns" due to lack of blinding and variability in outcome measures.

Conclusion:

Exercise-based interventions, especially cervical stabilization exercises, can significantly improve cervical proprioception in individuals with FHP. However, methodological heterogeneity and diagnostic inconsistencies limit definitive conclusions. Future studies should standardize diagnostic criteria, outcome assessments, and investigate long-term effects across diverse populations.

Keywords: proprioception, posture, cervical vertebrae, exercise therapy

Registration: International Prospective Register of Systematic Reviews ***

Introduction

Proprioception, the body's ability to sense its position, movement, and spatial orientation, is fundamental for motor control, postural stability, and injury prevention [1, 2]. In the cervical spine, proprioception plays a critical role in coordinating head and neck movements, maintaining balance, and stabilizing the head during dynamic activities [3, 4]. The cervical region is densely populated with muscle spindles and mechanoreceptors, making it highly reliant on accurate proprioceptive input for proper function [5]. Disruptions in cervical proprioception can lead to impaired joint position sense (JPS), increased joint position error (JPE), and a higher risk of musculoskeletal dysfunction [5, 6].

One of the most common postural deviations affecting cervical proprioception is forward head posture (FHP), characterized by anterior displacement of the head relative to the shoulders and an increased craniovertebral angle (CVA) [7]. FHP is prevalent across various populations, particularly among individuals with sedentary lifestyles, prolonged screen use, or occupations requiring sustained forward head positions [8]. The condition is associated with multiple adverse effects, including neck pain, muscle fatigue, reduced respiratory efficiency, and diminished quality of life [9-11]. Importantly, FHP has been linked to impaired cervical proprioception, as the altered alignment may disrupt afferent feedback from cervical muscle spindles and joint mechanoreceptors [12, 13]. Studies suggest that individuals with FHP exhibit greater JPE compared to those with normal posture, indicating diminished proprioceptive acuity [14, 15].

Despite growing recognition of the relationship between FHP and proprioceptive deficits, there remains a lack of consensus on diagnostic criteria for FHP. Various studies have proposed different CVA thresholds (e.g., ≤53°, <50°, <49°, or <48°), leading to inconsistencies in study populations and intervention outcomes [16-19]. Additionally, while exercise-based interventions[18, 20]—such as cervical stabilization exercises and postural correction—have shown promise in improving cervical proprioception, the optimal exercise prescription (frequency, intensity, type, and duration) remains unclear. Some studies suggest that proprioceptive training yields greater improvements than conventional strength training, while others highlight the benefits of combined approaches [21]. Furthermore, the mechanisms by which different exercises enhance proprioception in FHP—whether through neuromuscular re-education, enhanced muscle spindle sensitivity, or cortical adaptation—require further exploration.

This systematic review aims to synthesize existing evidence on the effectiveness of exercise-based interventions in improving cervical proprioception in individuals with FHP. By evaluating the methodologies, outcomes, and limitations of current studies, this review seeks to clarify which interventions are most effective and identify gaps for future research. The findings may inform clinical practice by guiding evidence-based rehabilitation strategies for individuals with FHP-related proprioceptive impairments.

Methods

Protocol and registration

The systematic review follows the Preferred Reporting Items for Systematic Reviews and Meta-Analyses (PRISMA) guideline [22] and was registered in the International Prospective Register of Systematic Reviews (PROSPERO) with registration number CRD42023488327.

Eligibility criteria

Following the search phase, AAN and KK independently conducted a thorough review of all retrieved titles and abstracts. The systematic review's study inclusion process was carefully structured using the PICOS (Population, Intervention, Comparison, Outcome, and Study Design) criteria [23], ensuring a methodical and transparent approach to selecting relevant studies (Table 1).

****Table 1 is here****

Search strategy

The search included the electronic databases Web of Science, PubMed, and Scopus, from inception to April 20, 2025, with two authors (KK and MALG) searching independently, with discrepancies resolved through discussion and, if needed, the opinion of a third author [24]. The search strategy used specific Mesh terms and text words/phrases, combined using Boolean operators (e.g.,(exercise OR "physical activity" OR training) AND (cervical OR neck OR head) AND ("proprioception" OR "sense of equilibrium" OR "equilibrium sense" OR "position sense" OR "posture sense" OR "sense of position" OR "kinesthesis" OR "joint position sense" OR "sense of resistance")). The specific search strategy for each database is presented in Table 2. This review had no restrictions on language, and Google Translate was used to interpret studies not published in English. Additionally, the reference lists of the included studies were screened, and a grey literature search was performed, including sources such as conference proceedings, dissertations, clinical trial registries, and preprint servers (e.g., medRxiv, bioRxiv). The retrieved studies were organized using Endnote, and any duplicate entries were removed. The Connected Papers website (https://www.connectedpapers.com/) was used to enhance the search for relevant research.

****Table 2 is here****

Data extraction

Two authors (SF and FE) collected information from retrieved papers, including study details (author, year of publication, location), study design, sample description (sample size, sex, age and craniovertebral angle), exercise characteristics, experiment group intervention program, control group intervention, cervical proprioception measures, assessment tool, main outcomes, conclusion, and risk of bias (ROBINS-I and RoB-2 overall judgment). If any important information was missing, the corresponding authors were contacted via email, with a maximum of three attempts made to obtain the necessary details.

Quality assessment

The risk of bias in the included randomized controlled trials (RCTs) was independently assessed by two reviewers (MALG and MALI) using the Cochrane Risk of Bias Tool (RoB-2) [25] across five domains: randomization process, deviations from intended interventions, missing outcome data, outcome measurement, and selection of reported results. Each domain was evaluated through signaling questions and judged as "low risk," "some concerns," or "high risk" of bias Discrepancies were resolved through discussion to ensure consistency. The randomization process was scrutinized for sequence generation and allocation concealment, while deviations from interventions examined protocol adherence and intention-to-treat analysis. Missing data were assessed for attrition rates and handling methods, and outcome measurement focused on assessor blinding and objectivity. Selective reporting was verified by comparing pre-specified and published outcomes. Results were visualized using the Robvis tool to generate traffic light plots, providing a clear representation of bias across studies in accordance with Cochrane guidelines.

Data synthesis

The study used narrative data synthesis and followed the PRISMA guidelines [22] to ensure thorough and transparent reporting, improving the validity of the results. Due to different outcome measures, a meta-analysis was not possible, so the review used a Synthesis Without Meta-Analysis (SWiM) approach, following the SWiM reporting guideline [26]. To provide specific evidence, effect sizes (ESs) were computed from reported means and standard deviations and classified based on Hopkins' scale as trivial (<0.2), small (0.2–0.6), moderate (0.6–1.2), large (1.2–2.0), very large (2.0-4.0), and nearly perfect (> 4.0) [25]. The ES for each study was computed using the following formula Cohen's *d*:

$$d = \frac{M_1 - M_2}{SD_{pooled}}$$

Where M_1 and M_2 represent the means of the groups being compared, and SD_{pooled} is the pooled standard deviation, calculated as:

$$SD_{pooled} = \sqrt{\frac{(n_1 - 1) \times SD_1^2 + (n_2 - 1) \times SD_2^2}{n_1 + n_2 - 2}}$$

Where n_1 and n_2 are the sample sizes and SD_1 and SD_2 are the standard deviations for each group. Following this method, the computed ESs were classified according to Hopkins' scale to describe the strength of the effects of exercise interventions on cervical proprioception. Additionally, prior to the data merging, the level of agreement between reviewers at each stage of the evaluation process was systematically assessed using Kappa (κ) statistics. The strength of agreement was categorized into distinct levels: poor (κ ≤ 0.20), fair (κ = 0.21–0.40), moderate (κ = 0.41–0.60), substantial (κ = 0.61–0.80), or near-perfect (κ = 0.81–0.99) [27].

Certainty of evidence assessment

The certainty of the evidence for each primary outcome was evaluated using the GRADE (Grading of Recommendations, Assessment, Development, and Evaluation) approach, as described in the Cochrane Handbook for Systematic Reviews of Interventions. The GRADE framework assesses evidence across five domains: risk of bias, inconsistency, indirectness, imprecision, and publication bias. Outcomes were rated as high, moderate, low, or very low certainty [28]. Since all included studies were randomized controlled trials, the starting level of certainty was high and downgraded as necessary based on the identified limitations.

Results

Study identification

Based on the PRISMA guidelines [22], the electronic databases search process initially retrieved 851 articles, but after removing duplicates, 155 (18.2%) studies were left for further screening based on the inclusion and exclusion criteria. The review focused on studies that investigated the effects of exercise-based interventions on cervical proprioception in individuals with forward head posture. A summary of the study progression and the rationale for exclusions at each phase is depicted in a PRISMA diagram (Figure. 1).

****Figure 1 is here****

Descriptive characteristics of the included studies

The nine studies included in this systematic review, published between 2018 and 2024, encompassed 367 participants (102 males [27.8%], 265 females [72.2%]) with FHP and were conducted across Iran (n=4), Korea (n=2), Thailand (n=1), and India (n=2). All studies utilized RCT designs with sample sizes ranging from 27 to 60 participants, featuring a mean age of 22.6 ± 2.1 years and a predominance of female participants in four studies [19, 24, 29]. Moreover, one study did not mention the sex of the participants [30]. Considerable variation existed in the diagnostic criteria for FHP, CVA thresholds ranging from <48° to ≤53° [16-19, 24, 29-31] and one study using a broader range of 40°-60° [32], highlighting a key methodological inconsistency across studies. The included studies investigated seven distinct exercise-based interventions for improving cervical proprioception in individuals with FHP. Cervical stabilization exercises represented the most common intervention (n=3/9), implemented in Parishan et al. (2021), Goo et al. (2024), and Lee et al. (2022). Other interventions included postural correction with muscle co-contraction and stretching [18], whole-body vibration training [17], backward walking on a treadmill [29], the National Academy of Sports Medicine corrective exercise protocol [24], cervical retraction combined with muscle energy techniques [32], and conventional physiotherapy incorporating hot packs, ultrasound therapy, and posture correction exercises [30]. Program durations ranged from single sessions to 8 weeks, with treatment frequencies varying between daily to 3-4 sessions per week. This diversity of approaches reflects the current variability in clinical practice for managing FHP-related proprioceptive deficits. Control conditions consisted

of no intervention (n=3) [18, 24, 29], sham exercises (n=1) [17], or were absent (n=5) [16, 19, 30-32]. Outcome measures were similarly diverse: five studies assessed Joint Position Error (JPE) using laser pointers or digital inclinometers [16, 18, 19, 29, 31], while four evaluated Joint Position Sense (JPS) through range-of-motion instruments or repositioning tests [17, 24, 30, 32]. Table 3 provides a summary of the specific characteristics and retrieved data of all nine studies.

****Table 3 is here****

Effect Sizes of Interventions on JPS and JPE

The calculated ESs for interventions targeting cervical proprioception demonstrated clinically meaningful improvements across all studies, though the magnitude varied by intervention type and outcome measure (JPS vs. JPE). For JPS outcomes, four studies showed ES values for flexion changes ranging from small to nearly perfect (ES = 0.33–4.55) [17, 24, 30, 32], while another four studies reported ES values for extension changes ranging from trivial to nearly perfect (ES = 0.11–4.52) [17, 24, 30, 32]. Three studies demonstrated moderate to very large ES values for right rotation changes (ES = 1.11–3.59) [24, 30, 32], and three others showed large to very large ES values for left rotation changes (ES = 1.98–2.98) [24, 30, 32]. Additionally, one study found very large ES values for right flexion changes (ES = 2.30) and left flexion changes (ES = 3.13) [30]. For JPE outcomes, two studies reported ES values for flexion changes ranging from trivial to very large (ES = 0.19–3.66) [16, 19], while two others showed ES values for extension changes ranging from trivial to small (ES = 0.12–0.58) [16, 19]. For the joint relocation test, two studies found moderate to large ES values (ES = 1.19–1.46) [29, 31]. Additionally, one study reported moderate ES values for right rotation changes (ES = 0.86) and left rotation changes (ES = 0.63) [18]. Figure 2 illustrates all the ESs of interventions on JPS and JPE.

****Figure 2 is here****

Quality assessment

All nine included RCTs were assessed for risk of bias using the RoB-2 tool (Figure 3), with each study exhibiting "some concerns" in the overall rating. Domain-specific analysis revealed that most studies (6/9) raised concerns in Domain 2 (bias due to deviations from intended interventions) [17-19, 30-32], while 6/9 showed issues in Domain 4 (bias in outcome measurement) [16, 17, 24, 29-31]. Domain 1 (randomization) was rated as low risk in 6 studies [16-19, 30, 31] and some concerns in 3 [24, 29, 32]. All studies demonstrated low risk in Domains 3 (missing outcome data) and 5 (selection of reported results) [16-19, 24, 29-32]. Inter-rater agreement was perfect ($\kappa = 1.0$), confirming consistent bias assessments across reviewers. The findings highlight methodological limitations, particularly in intervention adherence and outcome measurement, across the majority of included trials.

**** Figure 3 is here****

Certainty of the evidence

The certainty of the evidence was assessed using the GRADE approach. For the outcome of JPS, the certainty of evidence was rated as moderate, due to some concerns about inconsistency related to variability in interventions and measurement tools. For JPE, the certainty of evidence was rated as low, primarily due to inconsistency and imprecision caused by small sample sizes and heterogeneous outcome measures. A summary of findings is presented in Table 4.

**** Table 4 is here****

Discussion

This systematic review evaluated the effectiveness of exercise-based interventions in improving cervical proprioception among individuals with FHP. The findings suggest that various exercise modalities—including cervical stabilization exercises, postural correction, whole-body vibration, and muscle energy techniques—can enhance proprioceptive acuity, as measured by JPS and JPE. However, the heterogeneity in exercise protocols, and outcome measures underscores the need for cautious interpretation and highlights gaps for future research.

Key findings and mechanisms of improvement

The reviewed studies consistently demonstrated that exercise interventions led to clinically meaningful improvements in cervical proprioception. For instance, cervical stabilization exercises showed improvements in JPS with ESs ranging from 1.11 to 3.59 in rotation tasks, while flexion improvements ranged from 0.33 to 4.55, highlighting substantial proprioceptive gains. These exercises, the most frequently studied intervention (n = 3), were particularly effective in reducing JPE and enhancing JPS [16, 19, 31]. These exercises target deep cervical flexors and scapular stabilizers, which are often weakened in FHP, thereby restoring neuromuscular control and afferent feedback from muscle spindles and mechanoreceptors [5]. For example, Parishan et al. (2021) reported significant reductions in repositioning errors in flexion (ES= 1.12=3.66) after 4 weeks of stabilization training, suggesting that neuromuscular re-education plays a critical role in proprioceptive recovery [19]. The efficacy of these exercises can be attributed to several key physiological mechanisms:

Neuromuscular re-education: Stabilization exercises promote the reactivation of deep cervical muscles that are often underutilized in FHP [33, 34]. By restoring proper muscle activation patterns, these exercises enhance dynamic joint stability and reduce reliance on superficial muscles (e.g., sternocleidomastoid), which are prone to overuse and fatigue [35].

Enhanced muscle spindle sensitivity: The deep cervical flexors are richly innervated with muscle spindles, which play a critical role in proprioceptive acuity [5]. In FHP, prolonged lengthening of these muscles may lead to spindle desensitization [36]. Stabilization exercises, particularly those involving slow, controlled movements with an emphasis on precision (e.g., chin tucks with head nods), may increase spindle sensitivity by restoring optimal muscle length-tension relationships [37].

Alternative interventions

Emerging evidence suggests that proprioceptive rehabilitation for FHP may benefit from a diverse range of interventions beyond traditional cervical stabilization exercises. Whole-body vibration (WBV) has shown particular promise, with Salami et al. (2018) demonstrating immediate improvements in JPS (ESs: flexion= 0.33, extension= 0.11) following a single session [17]. This acute effect likely stems from WBV's unique ability to stimulate Ia afferent fibers through tonic vibration reflexes, temporarily enhancing muscle spindle sensitivity and facilitating cortical proprioceptive remapping [17, 38]. However, the clinical utility of WBV remains uncertain due to the absence of long-term follow-up data and standardized protocols for vibration parameters (frequency, amplitude, duration). Similarly innovative, backward walking has emerged as a potentially valuable intervention, with Mahmoudi et al. (2024) reporting a large effect on joint repositioning accuracy (ES = 1.19) following a 4-week program [29]. This approach appears to work through multiple mechanisms - it challenges the postural control system in novel ways that demand increased reliance on cervical proprioceptive inputs while simultaneously promoting co-activation of deep cervical flexors and scapular retractors [39, 40]. Despite these promising results, practical implementation barriers exist, particularly the requirement for specialized equipment and close supervision.

Complementary approaches that incorporate elements of postural re-education or muscle energy techniques have individually shown efficacy in improving cervical JPS and JPE. For instance, Jantoon and Uthaikhup (2020) demonstrated benefits from postural correction combined with muscle co-contraction and stretching (ESs: extension= 0.12, right rotation= 0.86, left rotation= 0.63) in the JPE [18], while Shah et al. (2022) reported improvements using cervical retraction (ESs: flexion= 3.05, extension= 4.52, right rotation= 3.59, left rotation= 2.97) and muscle energy techniques (ESs: flexion= 4.55, extension= 1.69, right rotation= 1.11, left rotation= 1.98) in the JPS [32]. These distinct but synergistic strategies may offer particular advantages for patients with FHP or myofascial pain by addressing both soft tissue limitations and neuromuscular control deficits.

While these alternative modalities expand the therapeutic toolkit for FHP-related proprioceptive impairment, their relative efficacy compared to conventional stabilization exercises remains unclear, highlighting the need for comparative effectiveness research and standardized protocol development.

Limitations and future directions

Across all included studies, none reported adverse effects of exercise-based interventions on proprioception, with 77.77% of measured outcomes demonstrating moderate-to-nearly perfect ES values [18, 19, 24, 29-32]. This consistency underscores the clinical relevance of proprioceptive training in managing FHP-related dysfunctions, despite methodological variability. However, the current evidence base for exercise interventions targeting cervical proprioception in FHP has several key limitations that warrant careful consideration. A primary concern is the substantial methodological heterogeneity across studies, particularly regarding diagnostic criteria and outcome measurement. The lack of consensus on FHP classification is particularly problematic,

with studies employing varying CVA thresholds ranging from <48° to ≤53°[16-19, 24, 29-31], and one study using an exceptionally broad range of 40-60°[32]. This diagnostic inconsistency has important clinical implications, as research by Titcomb et al. (2024) suggests that even a 5° difference in CVA measurement could lead to misclassification of approximately 20% of cases [8], potentially confounding study results and limiting their generalizability. Measurement variability extends to proprioception assessment as well, where studies utilized different tools including laser pointers and digital inclinometers to measure JPE. These instruments likely yield divergent values due to inherent measurement error differences, making cross-study comparisons challenging. Methodological quality concerns are further compounded by consistent risk of bias issues identified across all included studies. The near-universal absence of participant and assessor blinding represents a particularly notable limitation, with only Salami et al. (2018) incorporating a sham control condition in their whole-body vibration study [17]. This lack of blinding raises concerns about potential placebo effects and experimenter bias influencing outcomes. Attrition reporting was similarly problematic, with only three of nine studies adequately documenting dropout rates [18, 19, 32], while others either reported no attrition or failed to address this critical methodological detail entirely [16, 17, 24, 29-31]. These reporting gaps make it difficult to assess the true robustness of the findings. Population representation issues present additional constraints on the evidence base's generalizability. The studies exhibited a pronounced age bias, with a mean participant age of 22.6 years that effectively excludes older adults - a population known to experience age-related declines in proprioceptive acuity [41]. This limitation is particularly relevant given that FHP prevalence increases with age and may have different underlying mechanisms in older populations. Similarly, the marked sex imbalance (72% female participants across studies) raises questions about potential sex-specific effects that remain unexplored. No studies addressed possible hormonal influences on proprioception, such as menstrual cycle variations in joint laxity and neuromuscular control that could potentially mediate intervention outcomes in female participants. The GRADE assessment revealed moderate certainty for improvements in JPS and low certainty for JPE, primarily due to methodological heterogeneity, small sample sizes, and inconsistent outcome measures across studies. While the findings support the efficacy of exercise interventions, the limited certainty underscores the need for higher-quality RCTs with standardized protocols and larger, more diverse populations to strengthen clinical recommendations.

Conclusion

This systematic review demonstrates that exercise-based interventions, particularly cervical stabilization exercises, can improve cervical proprioception in individuals with forward head posture. Despite variability in exercise protocols and methodological limitations, the evidence supports the integration of targeted proprioceptive training into rehabilitation programs. Future research should prioritize standardized diagnostic criteria, optimal exercise parameters, and long-term outcomes to strengthen clinical recommendations.

References

1. Héroux ME, Butler AA, Robertson LS, Fisher G, Gandevia SC. Proprioception: a new look at an old concept. J Appl Physiol.;132(3):811-814. https://doi.org/10.1152/japplphysiol.00809.2021
2. Hosseini K, Mohammadian Z, Alimoradi M, Shabani M, Armstrong R, Hogg J, et al. The immediate effect of a balance wobble board protocol on knee and ankle joint position sense in female soccer players. Acta Gymnica. 2023;53(1). http://dx.doi.org/10.5507/ag.2023.011
3. Malmström E-M, Fransson P-A, Jaxmar Bruinen T, Facic S, Tjernström F. Disturbed cervical proprioception affects perception of spatial orientation while in motion. Exp Brain Res. 2017;235:2755-66. https://doi.org/10.1007/s00221-017-4993-5
4. Smith DL, Haug MJ, Walsh MS. The effect of posture on neck proprioception and head/neck stabilization in asymptomatic participants. J Can Chiropr Assoc. 2019;63(2):100.
5. Peng B, Yang L, Li Y, Liu T, Liu Y. Cervical proprioception impairment in neck pain-pathophysiology, clinical evaluation, and management: a narrative review. Pain Ther. 2021;10:143-64. https://doi.org/10.1007/s40122-020-00230-z
6. Stanton TR, Leake HB, Chalmers KJ, Moseley GL. Evidence of impaired proprioception in chronic, idiopathic neck pain: systematic review and meta-analysis. Phys Ther. 2016;96(6):876-87. https://doi.org/10.2522/ptj.20150241
7. Lin G, Zhao X, Wang W, Wilkinson T. The relationship between forward head posture, postural control and gait: a systematic review. Gait Posture. 2022;98:316-329. https://doi.org/10.1016/j.gaitpost.2022.10.008
8. Titcomb DA, Melton BF, Bland HW, Miyashita T. Evaluation of the craniovertebral angle in standing versus sitting positions in young adults with and without severe forward head Posture. Int J Exerc Sci. 2024;17(1):73-85. https://doi.org/10.70252/gdnn4363
9. Sedaghati P, Alghosi M, Hosseini F, Ghafouri M, Fallahi Farrash F, Parvaneh Sarand A. Correlation between sagittal plane curvature of the upright human spine and postural stability in active young adults: a cross-sectional study. Physical Treatments-Specific Physical Therapy Journal. 2024;14(4):303-310. http://dx.doi.org/10.32598/ptj.14.4.584.2
10. Nair SP, Panchabhai CS, Panhale V. Chronic neck pain and respiratory dysfunction: a review paper. Bull Fac Phys Ther. 2022;27(1):21. https://doi.org/10.1186/s43161-022-00078-8
11. Kapreli E, Vourazanis E, Billis E, Oldham J, Strimpakos N. Respiratory dysfunction in chronic neck pain patients. A pilot study. Cephalalgia. 2009;29(7):701-10. https://doi.org/10.1111/j.1468-2982.2008.01787.x
12. Lee M-Y, Lee H-Y, Yong M-S. Characteristics of cervical position sense in subjects with forward head posture. J Phys Ther Sci. 2014;26(11):1741-3. https://doi.org/10.1589/jpts.26.1741

13. Yong M-S, Lee H-Y, Lee M-Y. Correlation between head posture and proprioceptive function in the cervical region. J Phys Ther Sci. 2016;28(3):857-60. https://doi.org/10.1589/jpts.28.857
14. Bahar SF, Amir A, Nade M, Javad S. The evaluation of cervical position sense in forward head posture subjects and compares it with normal subjects. 2015;16(1):48-57.
15. Reddy RS, Alshahrani MS, Tedla JS, Dixit S, Asiri F, Al-Otaibi ML, et al. Comparison of cervical joint position sense in computer users with neck pain, forward head posture, asymptomatic and non-computer users–a cross-sectional study. Physikalische Medizin, Rehabilitationsmedizin, Kurortmedizin. 2023;33(04):227-34.
16. Goo BW, Oh JH, Kim JS, Lee MY. Effects of cervical stabilization with visual feedback on craniovertebral angle and proprioception for the subjects with forward head posture. Medicine (Baltimore). 2024;103(2):e36845. https://doi.org/10.1097/md.0000000000036845
17. Salami A, Roostayi MM, Naimi SS, Shadmehr A, Baghban AA. The immediate effects of whole body vibration on cervical joint position sense in subjects with forward head posture. Muscles Ligaments Tendons J. 2018;8(2):255-60.
18. Jantoon N, Uthaikhup S. Effects of an exercise program on cervical posture and sensorimotor function in persons with forward head posture. J Assoc Med Sci. 2020;53(3):62-71.
19. Parishan S, Far AA, Taghizadeh C, Khani MM. A comparative study of the immediate, late and lasting effect of kinesio taping and stabilization exercises on neck proprioception in people with forward head posture: A double-blinded randomized clinical trial. Koomesh. 2021;23(6):720-9.
20. Lee MY, Kim SG, Lee HY. The effect of cervical stabilization exercise on active joint position sense: A randomized controlled trial. J Back Musculoskelet Rehabil. 2016;29(1):85-8. https://doi.org/10.3233/bmr-150601
21. Blomgren J, Strandell E, Jull G, Vikman I, Röijezon U. Effects of deep cervical flexor training on impaired physiological functions associated with chronic neck pain: a systematic review. BMC Musculoskelet Disord. 2018;19:1-17. https://doi.org/10.1186/s12891-018-2324-z
22. Page MJ, McKenzie JE, Bossuyt PM, Boutron I, Hoffmann TC, Mulrow CD, et al. The PRISMA 2020 statement: an updated guideline for reporting systematic reviews. Int J Surg. 2021;88:105906. https://doi.org/10.1016/j.ijsu.2021.105906
23. Methley AM, Campbell S, Chew-Graham C, McNally R, Cheraghi-Sohi S. PICO, PICOS and SPIDER: a comparison study of specificity and sensitivity in three search tools for qualitative systematic reviews. BMC Health Serv Res. 2014;14(1):1-10. https://doi.org/10.1186/s12913-014-0579-0
24. Middel B, Van Sonderen E. Statistical significant change versus relevant or important change in (quasi) experimental design: some conceptual and methodological

problems in estimating magnitude of intervention-related change in health services research. Int J Integr Care. 2002;2:e15. https://doi.org/10.5334/ijic.65

25. Campbell M, McKenzie JE, Sowden A, Katikireddi SV, Brennan SE, Ellis S, et al. Synthesis without meta-analysis (SWiM) in systematic reviews: reporting guideline. BMJ. 2020;368. https://doi.org/10.1136/bmj.l6890

26. Cohen J. Weighted kappa: Nominal scale agreement provision for scaled disagreement or partial credit. Psychol Bull. 1968;70(4):213. https://doi.org/10.1037/h0026256

27. Guyatt GH, Oxman AD, Vist GE, Kunz R, Falck-Ytter Y, Alonso-Coello P, et al. GRADE: an emerging consensus on rating quality of evidence and strength of recommendations. BMJ. 2008;336(7650):924-6. https://doi.org/10.1136/bmj.39489.470347.AD

28. Abdolahzadeh M, Daneshmandi H. Effect of a corrective exercise program based on the national academy of sports medicine's protocol on forward head angle and cervical joint position sense in females with forward head posture. The Scientific Journal of Rehabilitation Medicine. 2023;11(6):878-91. http://dx.doi.org/10.32598/SJRM.11.6.3

29. Mahmoudi M, Hovanloo F, Shahpar FM, Barati A. A period of backwards walking affects the craniovertebral angle, proprioception, balance, and foot pressure on forward head posture of female students. J Bodywork Mov Ther. 2024;40:1844-51. https://doi.org/10.1016/j.jbmt.2024.10.040

30. Panihar U, Joshi S. Enhancing respiratory functions and cervical proprioception with myofascial release and feedback respiratory training in forward head posture: a pilot randomized controlled trial. Journal of Nature and Science of Medicine. 2024;7(4):276-83.

31. Lee S-H, Yoo B-H, Pyo H-S, Lee D, Hong J-H, Yu J-H, et al. Effects of cervical stabilization and scapular stabilization exercise on the proprioception and craniovertebral angle and upper trapezius muscle tone of people with forward head posture. J Korean Soc Phys Med. 2022;17(4):1-13. https://doi.org/10.13066/kspm.2022.17.4.1

32. Shah I, Kulkarni P, Kumar A. Assessment and Comparison between Cervical Retraction Exercise and Muscle Energy Technique on Cervical Joint Position Sense in Individuals with Forward Head Posture. International Journal of Science and Healthcare Research. 2022;7(1):199-208. https://doi.org/10.52403/ijshr.20220132

33. Yang S, Boudier-Revéret M, Yi YG, Hong KY, Chang MC, editors. Treatment of chronic neck pain in patients with forward head posture: a systematic narrative review. Healthcare(Basel). 2023;11(19):2604. https://doi.org/10.3390/healthcare11192604

34. Lauman ST, Anderson DI. A neuromuscular integration approach to the rehabilitation of forward head and rounded shoulder posture: Systematic review of literature. J Phys Med Rehabil. 2021;3(2):61-72. https://doi.org/10.33696/rehabilitation.3.021

35. Bolandian A, Alimohammadi I, Forough B. Effect of proprioceptive exercises along with ergonomic intervention on middle deltoid and serratus anterior fatigue in dentists with non-specific chronic neck pain. Int J Occup Hyg. 2021;13(4):313-23.
36. Matthews P, Stein R. The sensitivity of muscle spindle afferents to small sinusoidal changes of length. J Physiol 1969;200(3):723-43. https://doi.org/10.1113/jphysiol.1969.sp008719
37. Anderson BC. Cervical spine manipulation versus proprioceptive neuromuscular facilitation of the cervical spine in the treatment of mechanical neck pain: University of Johannesburg (South Africa); 2014.
38. Zhou C, Zhu L, Liu Z, Tong Y, Xu Y, Jiang L, et al. Whole body vibration training promotes proprioceptive pathway for the treatment of stress urinary incontinence in rats. Transl Androl Urol. 2024;13(5):657. https://doi.org/10.21037/tau-23-675
39. Park H-K, Park J. Effect of 2 weeks backward walking exercise on cervical angle and gait parameters in college students with forward head posture. J Korean Soc Integr Med. 2021;9(3):135-44. https://doi.org/10.15268/ksim.2021.9.3.135
40. Kamble S, Kothari P, Kale P, Joshi J, Kadam A, Palekar TJ. Effect of Backward Walking on Treadmill with Inclination in Individuals with Forward Head Posture: A Comparative Study. Journal of Datta Meghe Institute of Medical Sciences University. 2024;19(3):435-40. https://doi.org/10.4103/jdmimsu.jdmimsu_92_22
41. Şekeröz S, Aslan Telci E, Akkaya N. Effect of chronic neck pain on balance, cervical proprioception, head posture, and deep neck flexor muscle endurance in the elderly. Turkish Journal of Geriatrics. 2019;22(2):163-171

Tables

Table 1. Selection criteria for studies.

	Inclusion criteria	Exclusion criteria
**Population**	• Individuals with FHP without pain<br>• Individuals with ≥ 18 years old<br>• Any sex	Individuals with cervical pathology
**Intervention**	Any movement-based intervention	More than one intervention was compared at the same time
**Comparison**	Control group without exercise, sham exercise group, or condition for comparison	Not applicable
**Outcomes**	Investigate the effect of the intervention on joint position error related to cervical proprioception (e.g. joint position error)	No related outcome
**Study Design**	RCTs and non-RCTs	Single-group intervention; Case studies; Reviews.

Abbreviations: RCTs, randomized controlled trials; non-RCTs, non-randomized controlled trials

Table 2. Databases search strategies.

Database	Complete search strategy
**Web of Science**	exercise OR "physical activity" OR training (Topic) AND cervical OR neck OR head (Topic) AND "proprioception" OR "vestibular sense" OR "sense of equilibrium" OR "equilibrium sense" OR "labyrinthine sense" OR "position sense" OR "posture sense" OR "sense of position" OR "kinesthesis" OR "joint position sense" OR "sense of resistance" (Topic)
**PubMed**	((exercise[Title/Abstract] OR "physical activity"[Title/Abstract] OR training[Title/Abstract]) AND (cervical[Title/Abstract] OR neck[Title/Abstract] OR head[Title/Abstract])) AND ("proprioception"[Title/Abstract] OR "vestibular sense"[Title/Abstract] OR "sense of equilibrium"[Title/Abstract] OR "equilibrium sense"[Title/Abstract] OR "labyrinthine sense"[Title/Abstract] OR "position sense"[Title/Abstract] OR "posture sense"[Title/Abstract] OR "sense of position"[Title/Abstract] OR "kinesthesis"[Title/Abstract] OR "joint position sense"[Title/Abstract] OR "sense of resistance"[Title/Abstract])
**Scopus**	(TITLE-ABS-KEY (exercise OR "physical activity" OR training) AND TITLE-ABS-KEY (cervical OR neck OR head) AND TITLE-ABS-KEY ("proprioception" OR "vestibular sense" OR "sense of equilibrium" OR "equilibrium sense" OR "labyrinthine sense" OR "position sense" OR "posture sense" OR "sense of position" OR "kinesthesis" OR "joint position sense" OR "sense of resistance"))

Table 3. Characteristics of the included studies.

Study details	Design of study	Sample Description	Exercise characteristics	Control group Intervention	Cervical proprioception measures	Assessment tool	Main outcomes	Conclusion	Risk of bias
Salami et al. (2018) [17] Iran	RCT	N=30 individuals with FHP<br>Sex=18 males and 12 females<br>Age=21.4 ± 2.1 years<br>CVA= < 48º	D=1 session<br>F= NA<br>I= NA<br>T= NA<br>T= Whole body vibration	Holding the same position for the same time without any vibration.	JPS	Cervical range of motion instrument	A significant reduction (P < 0.05) in JPS absolute errors in 2 target angles (adapted head posture and 50% extension ROM) in the vibration group.	A relative improvement in cervical JPS can be achieved when adding additional sensory input from whole body vibration stimulus to the head and neck retraction exercise.	Some concerns
Jantoon and Uthaikhup (2020) [18] Thailand	RCT	N=53 individuals with FHP<br>Sex=18 males and 35 females<br>Age=21.8 ± 1.4 years<br>CVA= < 50º	D= 4 weeks<br>F= 7 times a week<br>I= NA<br>T= NA<br>T= Postural correction, muscle co-contraction, and muscle stretching	No exercise	JPE	A laser pointer attached to the head	JPEs (rotation to right and rotation to left) before and after the exercise program were significantly found in the experimental group (P < 0.05).	The 4-week exercise program was effective in improving the joint position sense.	Some concerns
Parishan et al. (2021) [19] Iran	RCT	N=43 individuals with FHP<br>Sex=43 females<br>Age=23.8 ± 3.3 years<br>CVA= < 49º	D= 4 weeks<br>F= 2 times a day and 3 times a week<br>I= NA<br>T= NA<br>T= Cervical stabilization exercise	NA	JPE	Digital inclinometer	A significant difference in repositioning error angles was found before and one month after the intervention (P < 0.05).	Stabilization exercise was effective for reducing neck repositioning error angle and improving proprioception in individuals with FHP.	Some concerns
Shah et al. (2022) [32] India	RCT	N=60 individuals with FHP<br>Sex=33 males and 27 females<br>Age=23.3 ± 1.5 years<br>CVA= 40°-60°	D= 2 weeks<br>F= 3 times a week<br>I= NA<br>T= NA<br>T= Cervical retraction exercise and muscle energy technique	NA	JPS	Head repositioning accuracy test	The study found a significant reduction in error in cervical JPS within the groups (P < 0.05) and between the groups (P < 0.05) for all physiological movements.	Cervical Retraction as well as Muscle Energy Technique can be used to improve Cervical JPS. However, Cervical Retraction is better as compared to Muscle Energy Technique.	Some concerns
Lee et al. (2022) [31] Korea	RCT	N= 27 individuals with FHP<br>Sex= 15 males and 12 females<br>Age= 21.3 ± 1.1 years<br>CVA= < 60º	D= 6 weeks<br>F= 3 times a week<br>I= NA<br>T= 30 minutes<br>T= Cervical stabilization and scapular stabilization exercises	NA	JPE	A laser pointer attached to the head	There was a significant difference after the training programs in head repositioning error (P < 0.05).	Cervical and scapula stabilization exercises were effective to improve neck proprioception.	Some concerns
Abdolahzadeh and Daneshmandi, (2023) [24] Iran	RCT	N= 30 individuals with FHP<br>Sex=30 females<br>Age=20.2 ± 1.7 years<br>CVA= > 46º	D=8 weeks<br>F=3 times a week<br>I= NA<br>T=30-70 minutes<br>T=NASM's protocol	No exercise	JPS	Head repositioning error test	Cervical JPS in the intervention group had a significant improvement compared to the control group (P ≤ 0.001).	Corrective exercises based on NASM's protocol seem to improve the cervical joint position sense.	Some concerns
Goo et al. (2024) [16] Korea	RCT	N=30 individuals with FHP<br>Sexr=18 males and 12 females<br>Age=23.3 ± 1.5 years<br>CVA= ≤ 53º	D= 4 weeks<br>F= 4 times a week<br>I= NA<br>T= 30 minutes<br>T= Cervical stabilization exercise	NA	JPE	Digital inclinometer	There was a significant difference after the intervention in extension (P <0.05).	The cervical stabilization exercise was effective for the proprioception of subjects.	Some concerns
Mahmoudi et al. (2024) [29] Iran	RCT	N=46 individuals with FHP<br>Sex= 46 females<br>Age= 22.8 ± 2.3 years<br>CVA= >38º and < 52º	D= 4 weeks<br>F= 4 times a week<br>I= Treadmill speed (2.4 km/h first two weeks then increased to 3.4 km/h)<br>T= 10 minutes<br>T= Backward walking on treadmill	No exercise	JPE	A laser pointer attached to the head	There was a significant difference after the training program in neck proprioception (P= 0.001).	Backward walking enhanced the proprioception in individuals with FHP.	Some concerns
Panihar and Joshi (2024) [30] India	RCT	N= 48 individuals with FHP<br>Sex= NA<br>Age= 26.1 ± 4.7 years<br>CVA= < 50º	D= 6 weeks<br>F= 3 times per week<br>I= NA<br>T= NA<br>T= Conventional treatment: hot pack, ultrasound therapy and posture correction exercises	NA	JPS	Head repositioning error test	There was a significant difference after the training program in head repositioning error (P < 0.001).	Conventional treatment improved the proprioception in individuals with FHP.	Some concerns

Abbreviations: CVA, craniovertebral angle; D, duration; F, frequency; FHP, forward head posture; I, intensity; JPE, joint position error; JPS, joint position sense; N, number; NA, not applicable; NASM, National Academy of Sports Medicine; RCT, randomized controlled trial; ROM, range of motion; T, time/type

Table 4. Summary of findings (GRADE).

Outcome	No. of studies	Participants	Effect estimate	Certainty (GRADE)	Reason for downgrade
JPS	4 RCTs	~168	Trivial to nearly perfect improvement (ES: 0.11–4.55)	●●○○ Low	Inconsistency + imprecision: diverse tools and interventions, small samples
JPE	5 RCTs	~199	Trivial to very large improvement (ES: 0.12–3.66)	●●○○ Low	Inconsistency + imprecision: diverse tools and interventions, small samples

Abbreviations: GRADE, Grading of Recommendations, Assessment, Development, and Evaluation; JPS, joint position sense; JPE, joint position error; RCTs, randomized controlled trials.

Certainty ratings: ●●●● High, ●●●○ Moderate, ●●○○ Low, ●○○○ Very low.

Figures

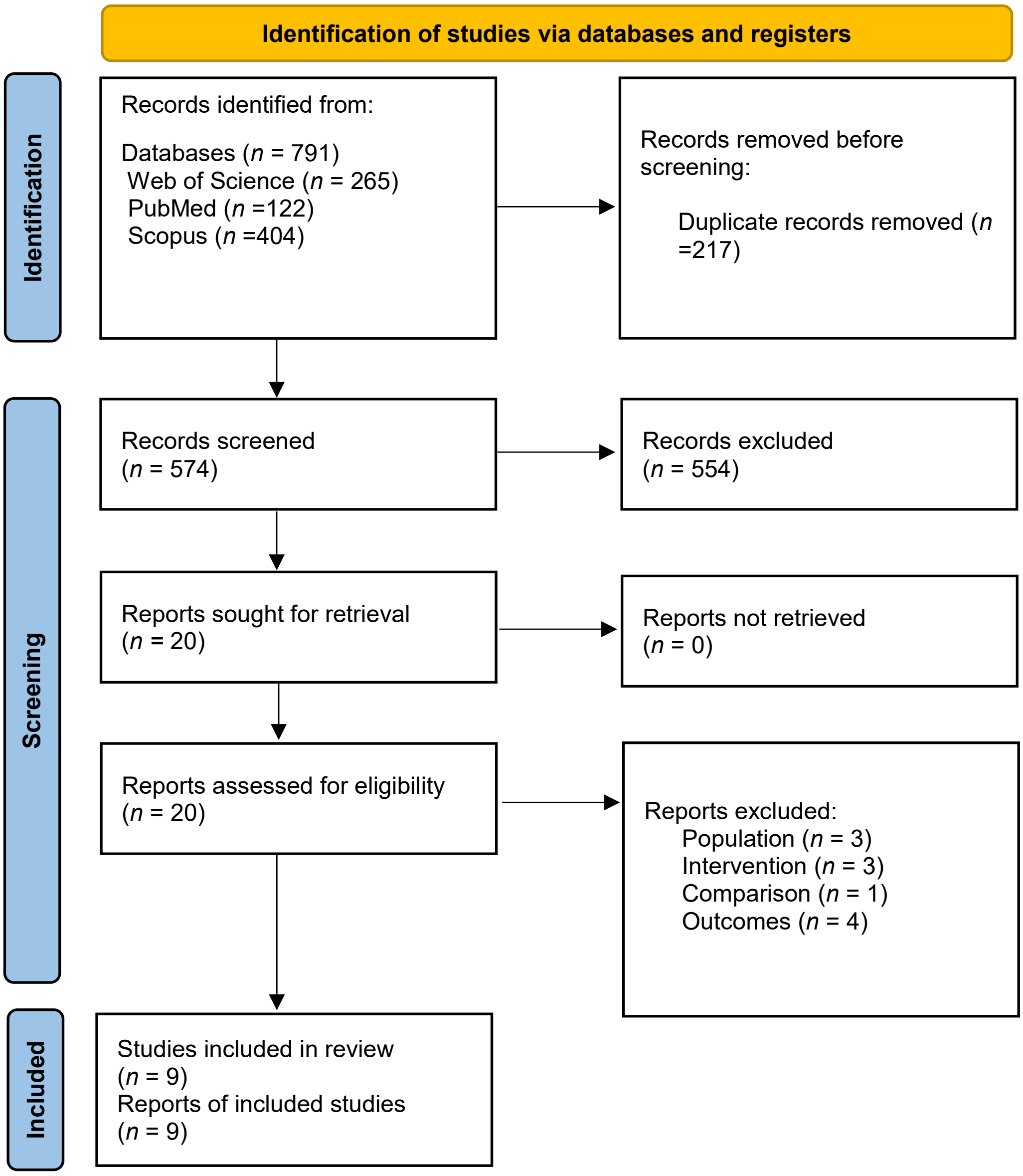

Figure 1. Preferred Reporting Items for Systematic Reviews and Meta-Analyses (PRISMA) 2020 flow diagram for new systematic reviews, including searches of databases and registers.

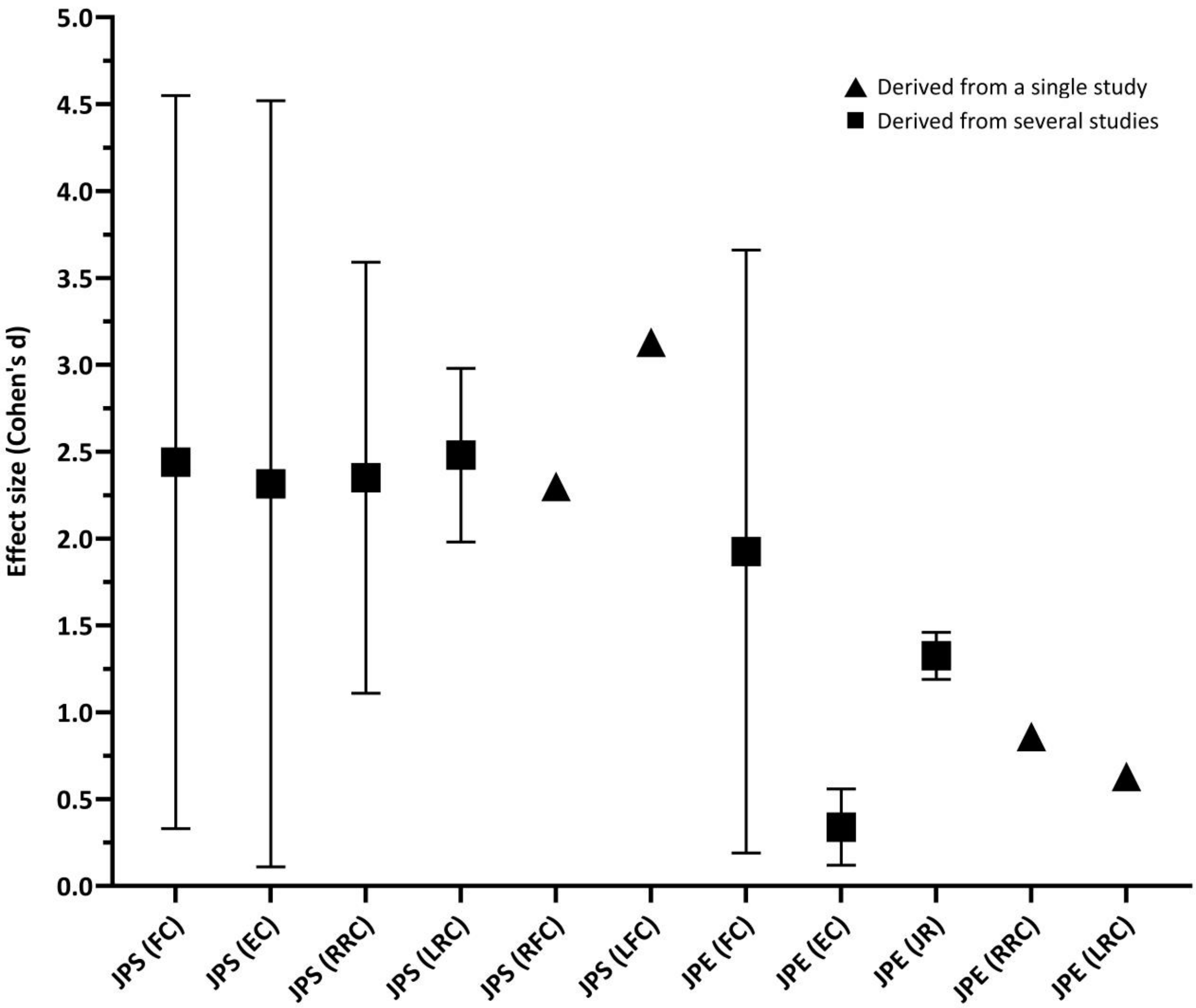

Figure 2. Effect Sizes of interventions on JPS and JPE. Abbreviations: JPS: joint position sense, JPE: joint position error, FC: flexion changes, EC: extension changes, RRC: right rotation changes, LRC: left rotation changes, RFC: right flexion changes, LFC: left flexion changes, and JR: joint relocation.

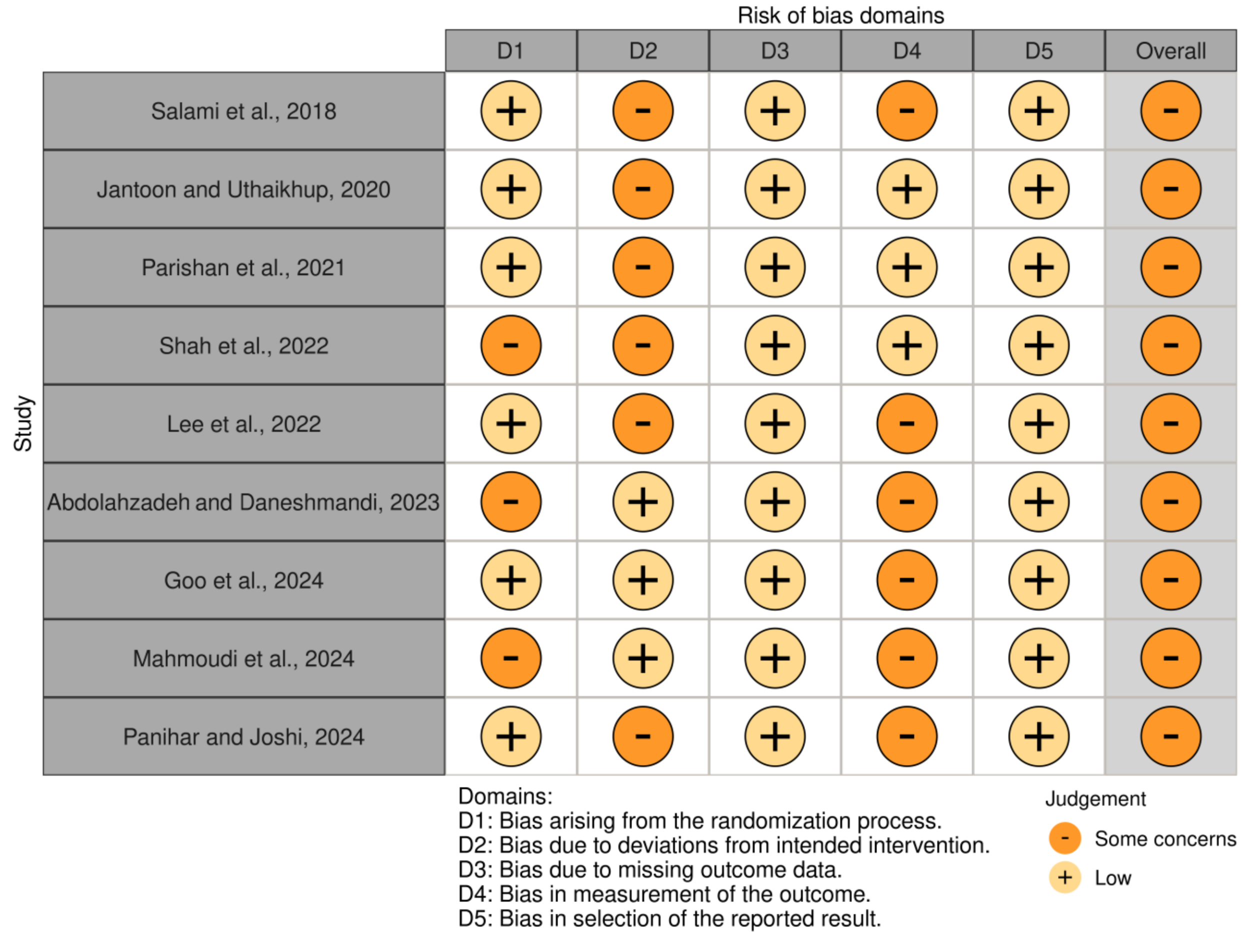

Figure 3. Risk of bias summary for randomized controlled trials evaluated with RoB-2.

Click here to access/download
Supporting Information
PRISMA_2020_checklist.docx
