# Supplementary material for: Exercise therapy to improve cervical proprioception in individuals with asymptomatic forward head posture: A systematic review of randomized controlled trials
Source: PLoS One. 2025 Sep 18;20(9):e0330665. doi: 10.1371/journal.pone.0330665 (PMC12445534; doi:10.1371/journal.pone.0330665)
Supplement: Attachment — Submitted filename: Response to Reviewers-July 9, 2025.docx [file pone.0330665.s004.docx]

Dear Reviewer,

Thank you for your valuable feedback on our manuscript titled "**Exercise therapy for cervical proprioception in individuals with asymptomatic forward head posture: a systematic review of randomized controlled trials**" We have carefully addressed your comments and made significant revisions to improve the quality of our work. Below, you will find our detailed responses to each of your suggestions. We appreciate your insights and look forward to your thoughts on the revised manuscript.

Best regards,

**# Reviewer 1**

**1. Unclear Definition of 'Diagnostic Inconsistencies': Mentioning "diagnostic inconsistencies" is appropriate, but without a brief example (e.g., varying CVA thresholds), readers are left in the dark.**

**Response:** Thank you for your feedback. We have clarified the phrase "diagnostic inconsistencies" by adding an example to improve clarity. Here is the revised text (lines 67 to 69):

“However, methodological heterogeneity and diagnostic inconsistencies—such as variations in craniovertebral angle thresholds used to define FHP—limit the ability to draw definitive conclusions”

**2. The phrase “exercise-based interventions” is repeated excessively.**

**Response:** Thank you for your observation. We have revised the manuscript to reduce redundancy by rephrasing and varying the terminology used in place of "exercise-based interventions" where appropriate (e.g., "exercise programs," "training interventions," or simply "interventions").

**3. The paper states that exercise may improve proprioception via “neuromuscular re-education” but does not adequately elaborate.**

**Response:** Thank you for your valuable feedback. We have expanded the discussion to elaborate on how neuromuscular re-education contributes to improved proprioception in individuals with FHP. Specifically, we now detail how stabilization exercises retrain deep cervical muscle activation, restore optimal spindle sensitivity, and promote cortical adaptations that enhance proprioceptive integration. Here is the revised text (lines 306 to 314):

“Neuromuscular re-education: Neuromuscular re-education refers to the process of retraining the nervous system to improve the coordination and activation of muscles, particularly those responsible for postural control and joint stability. In the context of FHP, prolonged anterior displacement of the head leads to overactivity of superficial muscles (e.g., sternocleidomastoid and upper trapezius) and underactivity of deep cervical flexors (e.g., longus colli and longus capitis). Stabilization exercises target these deep muscles, promoting their reactivation and restoring optimal muscle activation patterns. This re-education process enhances dynamic joint stability and reduces reliance on superficial muscles, which are prone to fatigue and inefficient for proprioceptive feedback.”

**4. There is no mention of how the lack of blinding in included RCTs may affect bias in outcome measurements.**

**Response:** Thank you for your insightful comment. We have now addressed this issue in the “Limitations and future directions” section. Specifically, we discuss how the lack of blinding in most included studies may have introduced performance and detection bias, particularly given the subjective nature of proprioception outcomes. We have also highlighted the need for future studies to implement assessor blinding and credible sham controls to enhance methodological rigor.

Here is the revised text:

“An important methodological limitation observed across most included studies was the lack of blinding of participants and/or outcome assessors. Given that proprioception outcomes such as JPE and JPS are partially reliant on subjective interpretation and participant cooperation, the absence of blinding can introduce performance and detection biases. Without proper blinding, participants may respond differently due to expectations about the intervention (placebo effects), and assessors may unconsciously influence measurements or data interpretation. This could inflate the perceived effectiveness of the interventions. Only one study attempted a sham control condition to mitigate this bias[17]. Therefore, future trials should incorporate assessor blinding and, where feasible, participant blinding or credible sham controls to improve internal validity.”

**5. While ESs are given per direction (flexion, extension), this becomes hard to digest. A table summarizing overall ESs by intervention type (e.g., stabilization vs. WBV) and direction would help.**

**Response:** Thank you for your suggestion. To address your comment a new Table was added to manuscript.

Here is the added Table:

**Table 4.** Summary of effect sizes by intervention type.

| **Intervention** | **Measurement** | **ES range** | **Magnitude** | **Studies** |
| --- | --- | --- | --- | --- |
| Cervical stabilization exercise | Flexion | 0.19-3.66 | Trivial to very large | Parishan et al. and Goo et al |
|  | Joint relocation | 1.40 | large | Lee et al. |
| Scapular stabilization exercise | Joint relocation | 1.46 | large | Lee et al. |
| Postural correction | Extension | 0.12 | Trivial | Jantoon and Uthaikhup |
|  | Right rotation | 0.86 | Moderate |  |
|  | Left rotation | 0.63 | Moderate |  |
| WBV | Flexion | 0.33 | Small | Salami et al. |
|  | Extension | 0.11 | Trivial |  |
| Backward walking on treadmill | Joint relocation | 1.19 | Moderate | Mahmoudi et al. |
| NASM | Flexion | 2.14 | Very large | Abdolahzadeh and Daneshmandi |
|  | Extension | 0.87 | Moderate |  |
|  | Right rotation | 2.02 | Very large |  |
|  | Left rotation | 2.23 | Very large |  |
| Cervical retraction combined with muscle energy techniques | Flexion | 3.05-4.55 | Very large to nearly perfect | Shah et al. |
|  | Extension | 1.69-4.52 | Large to nearly perfect |  |
|  | Right rotation | 1.11-3.59 | Moderate to very large |  |
|  | Left rotation | 1.98-2.97 | Large to very large |  |
| Conventional physiotherapy | Flexion | 1.69 | Large | Panihar and Joshi |
|  | Extension | 2.48 | Very large |  |
|  | Right rotation | 3.44 | Very large |  |
|  | Left rotation | 2.98 | Very large |  |
|  | Right flexion | 2.30 | Very large |  |
|  | Left Flexion | 3.13 | Very large |  |

**Abbreviations:** ES, effect size; WBV, whole body vibration; NASM, National Academy of Sports Medicine.

**6. Statements like “more research is needed” or “future studies should standardize protocols” are expected and lack depth.**

**Response:** Thank you for your insightful comment. To address your comment, we revised the conclusion of the manuscript. Here is the revised paragraph:

“This systematic review demonstrates that cervical stabilization exercises may lead to improvements in cervical proprioception, likely through neuromuscular re-education of deep cervical flexors. To translate these findings into clinical practice, future research should focus on three key areas: (1) establishing standardized diagnostic criteria using specific CVA thresholds for FHP and optimizing exercise prescription, (2) developing optimized intervention protocols incorporating objective adherence monitoring through wearable sensors, and (3) expanding study populations to include older adults and high-risk occupational groups while stratifying outcomes by age, sex, and comorbidities. Addressing these priorities will enhance intervention reproducibility and improve clinical applicability for diverse populations with FHP-related proprioceptive deficits.”

**# Reviewer 2**

**1. The title should include "systematic review of randomized controlled trials" In addition, provide direction in the title.**

**Response:** Thank you for your comment. The title was revised based on your recommendation.

Here is the revised title:

“Exercise therapy to improve cervical proprioception in individuals with asymptomatic forward head posture: a systematic review of randomized controlled trials”

**2. Why did you not include google scholar?**

**Response:** Thank you for your question. The Google Scholar was used as grey literature to identify further pertinent studies. Here is the revised section:

“Additionally, the reference lists of the included studies were screened, and a grey literature search was conducted using Google Scholar to identify further relevant studies, including conference proceedings, dissertations, clinical trial registries, and preprint servers (e.g., medRxiv, bioRxiv).”

**3. Can this be the case because it is the most widely used intervention?**

**Response:** Thank you for your valuable feedback. We have revised the text to clarify that cervical stabilization exercises are the most widely studied—rather than definitively the most effective—intervention for FHP, acknowledging limitations in the current evidence. Here is the revised text:

“Current evidence suggests cervical stabilization exercises, the most studied intervention for FHP, may improve cervical proprioception. However, methodological heterogeneity and diagnostic inconsistencies, such as variations in craniovertebral angle thresholds used to define FHP, limit the ability to draw definitive conclusions.”

**4. You can also add this citation in here to diversify your references:**

**Ouattas, A., Wellsandt, E., Hunt, N. H., Boese, C. K., & Knarr, B. A. (2019). Comparing single and multi-joint methods to detect knee joint proprioception deficits post primary unilateral total knee arthroplasty. Clinical Biomechanics, 68, 197-204.**

**Response:** Thank you. It was added.

**5. What about threshold to detect passive motion?**

**Response:** Thank you for your comment. The highlighted sentence was revised.

Here is the revised sentence:

“Disruptions in cervical proprioception can lead to impaired joint position sense (JPS), increased joint position error (JPE), and a higher risk of musculoskeletal dysfunction, reduced threshold to detect passive motion, and a higher risk of musculoskeletal dysfunction.”

**6. Do you mean decreased angle in here?**

**Increase in CVA leads to more upward head while decrease in CVA leads to more forward and downward head**

**Response:** Thank you for your comment. It was revised.

Here is the revised sentence:

“One of the most common postural deviations affecting cervical proprioception is forward head posture (FHP), characterized by anterior displacement of the head relative to the shoulders and an decreased craniovertebral angle (CVA)”

**7. Reduced not diminished. Diminished portrays almost vanishing.**

**Response:** Thank you. It was revised. Here is the revised sentence:

“The condition is associated with multiple adverse effects, including neck pain, muscle fatigue, reduced respiratory efficiency, and reduced quality of life.”

**8. Impaired? The cited papers did not investigate proprioception impairments in clinical populations. Please revise. This should be changed to: "FHP linked to reduced proprioception".**

**Response:** Thank you for your comment. Here is the revised sentence:

“Importantly, FHP has been linked to reduced cervical proprioception, as the altered alignment may disrupt afferent feedback from cervical muscle spindles and joint mechanoreceptors”

**9. What is this abbreviation? Please make sure you define abbreviations first.**

**Response:** Thank you. The abbreviation has been mentioned before at line 91.

**10. Again, be careful of the word choice. This should be changed to reduced not diminished.**

**Response:** Thank you for your recommendation. Here is the revised sentence:

“Studies suggest that individuals with FHP exhibit greater JPE compared to those with normal posture, indicating reduced proprioceptive acuity.”

**11. The discussion could benefit from a subsection that specifically addresses the exercise prescription (i.e., frequency, intensity, type, and duration); specifically, since the authors stated in the introduction that exercise prescription remains unclear and is a clear limitation within the literature.**

**Response:** Thank you for your comment. Here is the new section in the Discussion:

“Analysis of the included studies (Table 3) reveals variability in exercise prescription parameters for improving cervical proprioception in FHP. While cervical stabilization exercises (3-4 sessions/week for 4-6 weeks) emerged as the most consistently effective intervention (ES range: 0.19-3.66), critical gaps remain in our understanding of optimal dosing. Only one study specified exercise intensity (backward walking at 2.4-3.4 km/h), while others lacked objective intensity measures, and none compared different frequencies or durations head-to-head. Alternative approaches like whole-body vibration (single session) and muscle energy techniques (2weeks) showed promise but require longer-term study. Notably, none of the 9 studies compared different frequencies or durations directly, and only 4/9 specified session duration (10–70 minutes).  This heterogeneity in frequency (daily to 7×/week), duration (single session to 8 weeks), and unspecified intensities underscores the need for standardized protocols and comparative effectiveness research to establish evidence-based prescription guidelines.”

**12. What about Randomized Control Trials? You don't seem to mention it here.**

**Response:** Thank you for your comment. In Table 1, we separated inclusion and exclusion criteria which also included the type of studies. Below you see the Table 1:

|  | **Inclusion criteria** | **Exclusion criteria** |
| --- | --- | --- |
| **Population** | Individuals with FHP without pain  Individuals with ≥ 18 years old  Any sex | Individuals with cervical pathology |
| **Intervention** | Any movement-based intervention | More than one intervention was compared at the same time |
| **Comparison** | Control group without exercise, sham exercise group, or condition for comparison | Not applicable |
| **Outcomes** | Investigate the effect of the intervention on joint position error related to cervical proprioception (e.g. joint position error) | No related outcome |
| **Study Design** | RCTs and non-RCTs | Single-group intervention; Case studies; Reviews. |

**Table 1.** Selection criteria for studies.

**Abbreviations:** RCTs, randomized controlled trials; non-RCTs, non-randomized controlled trials.

**13. March 28 or April 20? Make sure you have your dates matching and correct.**

**Response:** Thank you. Here is the revise sentence:

“The search included the electronic databases Web of Science, PubMed, and Scopus, from inception to April 20, 2025, with two authors (KK and MALG) searching independently, with discrepancies resolved through discussion and, if needed, the opinion of a third author.”

**I do appreciate the motive behind this review manuscript, which is focused on summarizing the current unknowns, and potential benefits of exercise-based interventions in improving cervical proprioception in individuals with forward head posture. This is a well-flowed review article whose theme is suitable to this journal. The objectives are relevant, and the discussion is well-written and flows with the paper. However, the authors overlooked several key and relevant literature that deems this systematic review incomplete and requires major revisions. Mainly since the reviewers did not include “google scholar” as one of the search engines. By running a search using “google scholar”, I found several key RCT publications that could play a major role on the evidence provided in this systematic review, which are provided bellow. The authors also misused several words which portrays a completely different meaning. The manuscript requires major revisions for vocabulary and grammatic errors. Lastly, the discussion could benefit from a subsection that specifically addresses the exercise prescription (i.e., frequency, intensity, type, and duration); specifically, since the authors stated in the introduction that exercise prescription remains unclear and is a clear limitation within the literature. Once these points, and all the other revisions mentioned in the article, are addressed, I will indicate this systematic review research article for publication.**

**1. Miçooğulları, M., Yüksel, İ., & Angın, S. (2024). Efficacy of scapulothoracic exercises on proprioception and postural stability in cranio-cervico-mandibular malalignment: A randomized, double-blind, controlled trial. Journal of Back and Musculoskeletal Rehabilitation, 37(4), 883-896.
2. Kang, N. Y., Im, S. C., & Kim, K. (2021). Effects of a combination of scapular stabilization and thoracic extension exercises for office workers with forward head posture on the craniovertebral angle, respiration, pain, and disability: A randomized-controlled trial. Turkish journal of physical medicine and rehabilitation, 67(3), 291–299. https://doi.org/10.5606/tftrd.2021.6397
3. Sarıyıldız, A., Deniz, V., & Başaran, S. (2022). Effectiveness of corrective exercise program on alignment, muscle activation and biomechanical properties in forward head posture: a randomized controlled trial. Cukurova Medical Journal, 49(4), 1082-1092.
4. Igbokwe, E. O., Taube, W., & Beinert, K. (2022). A Comparison of the Effects of Stochastic Resonance Therapy, Whole-Body Vibration, and Balance Training on Pain Perception and Sensorimotor Function in Patients With Chronic Nonspecific Neck Pain: Protocol for a Randomized Controlled Trial. JMIR Research Protocols, 11(6), e34430.
5. Gulati, M., Harikesavan, K., & Venkatesan, P. (2025). Immediate effects of thoracic postural correction taping on sensorimotor and respiratory functions in healthy office workers with forward head posture-A randomized controlled trial. Journal of Bodywork and Movement Therapies.
6. Anwar, S., Zahid, J., Alexe, C. I., Ghazi, A., Mareș, G., Sheraz, Z., ... & Gasibat, Q. (2024). Effects of Myofascial Release Technique along with Cognitive Behavior Therapy in University Students with Chronic Neck Pain and Forward Head Posture: A Randomized Clinical Trial. Behavioral Sciences, 14(3), 205.
7. Sarıyıldız, A., Deniz, V., & Başaran, S. (2022). Effectiveness of corrective exercise program on alignment, muscle activation and biomechanical properties in forward head posture: a randomized controlled trial. Cukurova Medical Journal, 49(4), 1082-1092.
8. Dareh-Deh, H. R., Hadadnezhad, M., Letafatkar, A., & Peolsson, A. (2022). Therapeutic routine with respiratory exercises improves posture, muscle activity, and respiratory pattern of patients with neck pain: A randomized controlled trial. Scientific reports, 12(1), 4149.**

**Response:** We appreciate this observation. While we carefully evaluated all identified articles, they could not be included as did not meet our predefined PICOS criteria (Table 1). Additionally, our preliminary grey literature search of Google Scholar prior to the systematic review did not yield any eligible studies that satisfied our inclusion parameters. Here are the reasons for exclusion of the articles you mentioned, and as a result, we did not include them in our review:

1. In the study by **Miçooğulları et al. (2024),** the inclusion criteria specified that participants had temporomandibular joint dysfunction and used two questionnaires related to this condition. This is the reason for excluding this study.
2. In the study by **Kang et al. (2021),** participants experienced pain, whereas our focus was on asymptomatic FHP. Therefore, this study was excluded.
3. The study by **Sarıyıldız et al. (2022)** did not include any variables related to proprioception, which is why it was not included.
4. The study by **Igbokwe et al. (2022)** involved participants with chronic nonspecific neck pain, leading to its exclusion.
5. In the study by **Gulati et al. (2025),** taping was used as the intervention, and since this is not a movement-based intervention, it was excluded.
6. The study by **Anwar et al. (2024)** included participants with chronic neck pain, which is why it was not included.
7. The study you mentioned is the same as the one by **Sarıyıldız et al. (2022) (3^rd^ study you mentioned),** with the same title and authors, and was therefore excluded.
8. In the study by **Dareh-Deh et al. (2022),** participants had neck pain, leading to its exclusion.

**# Reviewer 3**

**1. As the authors mentioned in the introduction, one of the purposes of this study was to clarify which interventions are most effective among different methods. Therefore, I recommend adding a new figure that summarizes the effect sizes of each intervention method. This visualization would help the readers to interpret the authors’ claims.**

**Response:** We appreciate your insightful comment. In response to Reviewer 2’s suggestion, we have created Table 4 to summarize the effects of different intervention types. We believe this table effectively addresses your concerns by providing a clear overview of the comparative effectiveness across interventions.

**2. Regarding alternative interventions in the discussion, where it states, “While these alternative modalities …, their relative efficacy compared to conventional stabilization exercises remains unclear (line 342-344 of page 9)”. Indeed, this claim is important to reveal the challenges for the alternative interventions, but the rationale behind this claim is unclear. Please clarify the reason why the effectiveness of alternative intervention methods is unknown compared to traditional interventions for the readers.**

**Response:** We thank the reviewer for their valuable feedback highlighting the importance of clarifying the reasoning behind the statement concerning the ambiguous relative efficacy of alternative interventions in comparison to traditional stabilization exercises. The primary reason for this uncertainty is the paucity of rigorous, direct comparative studies that evaluate alternative modalities against traditional cervical stabilization in a standardized manner. Additionally, existing research often exhibits considerable variability in intervention protocols and outcome measures, coupled with a predominant focus on short-term effects rather than long-term clinical outcomes. These factors collectively limit the ability to draw definitive conclusions about the comparative effectiveness of these interventions. In response to your comment, we have revised the manuscript to specifically address these issues and improve clarity for the readers. Here is the revised paragraph:

“While these alternative modalities expand the therapeutic options for addressing proprioceptive impairments associated with FHP, their relative efficacy compared to conventional cervical stabilization exercises remains unclear. This uncertainty primarily arises from the limited number of high-quality randomized controlled trials directly comparing these interventions, the heterogeneity in study designs, and the lack of standardized treatment protocols regarding dosage, frequency, and duration. Furthermore, many studies focus on short-term outcomes without sufficient long-term follow-up, hindering the ability to determine sustained clinical benefits.”

**3. As pointed out above, the present discussion regarding the comparison of intervention methods seems vague. Therefore, it is unclear which results support a part of the conclusion (“… particularly cervical stabilization exercises, can improve …“, lines 387-388 of page 10). Specifically, a question is: if this conclusion that cervical stabilization exercises were most effective is based on effect size, should the authors consider the fact that the cervical retraction method also had equivalent effect sizes?**

**Response:** We appreciate the reviewer's valuable comment regarding the comparison of intervention methods and the conclusion about cervical stabilization exercises. While cervical retraction combined with muscle energy techniques demonstrated large effect sizes in one study (Shah et al., 2022), our conclusion highlighting cervical stabilization exercises is based on their consistent effectiveness across multiple studies (3 of 9 included trials) and their mechanistic focus on neuromuscular re-education of deep cervical flexors, which are particularly relevant for FHP-related proprioceptive deficits. We have refined the conclusion to more precisely reflect that stabilization exercises are the most studied intervention with replicated benefits, while acknowledging that other modalities like cervical retraction show promise but require further validation. This clarification maintains our original findings while addressing the reviewer's concern about comparative effectiveness. Here is the revised conclusion:

“This systematic review demonstrates that current evidence suggests cervical stabilization exercises, the most studied intervention for FHP, may improve cervical proprioception, likely through neuromuscular re-education of deep cervical flexors. While other modalities like cervical retraction show promise, further research is needed to compare efficacy across interventions. To translate these findings into clinical practice, future research should focus on three key areas: (1) establishing standardized diagnostic criteria using specific CVA thresholds for FHP and optimizing exercise prescription, (2) developing optimized intervention protocols incorporating objective adherence monitoring through wearable sensors, and (3) expanding study populations to include older adults and high-risk occupational groups while stratifying outcomes by age, sex, and comorbidities. Addressing these priorities will enhance intervention reproducibility and improve clinical applicability for diverse populations with FHP-related proprioceptive deficits.”

**4. Regarding the abstract, there is a concern regarding the claim: “Cervical stabilization exercises demonstrated the most consistent benefits, particularly in rotation and flexion tasks” (lines 62-63 of page 2). There are two questions: Firstly, what is this “most” compared with (directions of motion or intervention methods)? Second, if the comparison is regarding intervention methods, what is the logic of this claim? If the claim is based on the number of studies (i.e., the fact that 3/9 are stabilization exercises), it could be misunderstood that alternative intervention methods lacked consistency. To ensure rigor, it is helpful to clarify that consistency regarding alternative interventions could not be evaluated due to the number of studies.
Together, regarding both the conclusion and abstract, the authors might want to consider clarifying the reasons for their claim that stabilization exercises were most or consistently effective. I recommend giving the evidence explicitly in the Discussion. If there is no valid basis, I would recommend that the authors exclude the claim on which interventions are most effective from the conclusions and the result of abstract.**

**Response:** We thank the reviewer for this important point. The statement regarding cervical stabilization exercises was based on the relatively consistent positive outcomes reported across studies of this intervention. However, we acknowledge that the limited number and heterogeneity of studies on alternative interventions prevent a robust comparison of consistency or effectiveness. To avoid misunderstanding, we have revised the abstract and discussion to clarify that while cervical stabilization exercises show promising results, the evidence is insufficient to definitively compare their efficacy with alternative methods.

**5. Regarding data synthesis and narrative review in the results, the authors grouped the studies that used standard outcome measures as JPE and otherwise as JPS. If this understanding is correct, there is a question about whether it is necessary to separate the included studies into two groups. If the authors interpret that both outcome measures are used to measure joint position sense (i.e., proprioception acuity), the authors could analyze all studies in a unified way without grouping. What are the criteria and rationale for separating JPE and JPS in this analysis?**

**Response:** We appreciate the reviewer’s insightful question regarding the separation of studies into JPE and JPS groups in our analysis. The rationale for this approach lies in the conceptual and methodological differences between the two measures: JPE quantifies the accuracy of repositioning the head to a target position (reflecting dynamic proprioceptive acuity), while JPS assesses the perception of joint position without movement (often involving passive motion detection). These distinct aspects of proprioception may respond differently to interventions, and combining them could obscure intervention effects due to variability in measurement tools (e.g., laser pointers for JPE vs. inclinometers for JPS) and task demands. Additionally, separating JPE and JPS aligns with clinical practice, as JPE is more directly tied to functional neuromuscular control in conditions like FHP, whereas JPS provides insight into sensory discrimination. This approach also follows conventions in existing literature, ensuring comparability with prior research. By analyzing them separately, we enhance the precision of our conclusions and offer clearer guidance for clinicians targeting specific proprioceptive deficits. We have clarified this rationale in the revised manuscript to improve transparency. Thank you for the opportunity to address this important methodological consideration.

**6. Regarding the conclusion, where it states, "… exercise-based interventions ...can improve cervical proprioception in individuals with forward head posture" (lines 387-389 of page 10). Indeed, the results of this review support the improvement of sensorimotor control using cervical spine movements (i.e., the improvement of “Joint Position Error Test”). However, it appears difficult to identify the mechanism of improvement, as the authors mention it in the Key findings and mechanisms of improvement, too. Therefore, to avoid misleading, the authors might want to consider avoiding a definitive claim that "can improve cervical proprioception."**

**Response:** Thank you for your comment. Here is the revised Conclusion:

“This systematic review demonstrates that cervical stabilization exercises may lead to improvements in cervical proprioception, likely through neuromuscular re-education of deep cervical flexors. To translate these findings into clinical practice, future research should focus on three key areas: (1) establishing standardized diagnostic criteria using specific CVA thresholds for FHP and optimizing exercise prescription, (2) developing optimized intervention protocols incorporating objective adherence monitoring through wearable sensors, and (3) expanding study populations to include older adults and high-risk occupational groups while stratifying outcomes by age, sex, and comorbidities. Addressing these priorities will enhance intervention reproducibility and improve clinical applicability for diverse populations with FHP-related proprioceptive deficits.”

**7. Page 2 Abstract and Page 4 Search strategy: The authors state “A systematic search of PubMed, Scopus, and Web of Science was conducted up to March 28, 2025” in Abstract-Methods (line 54 of page 2). However, the authors also state, “The search included the electronic databases Web of Science, PubMed, and Scopus, from inception to April 20, 2025…” in Search strategy (lines 132-133 of page 4). Does each statement indicate something different? In addition, did the authors have any restrictions on the publication year of studies they searched?**

**Response:** Thank you for your comment. The date of search strategy was revised and it was from inception to April 20, 2025. Also, there was no restriction on the publication year of studies (from inception …).

**8. Quality assessment: At the domain of the randomization process, were baseline differences between groups evaluated (if available)?**

**Response:** The RoB-2 tool does not directly assess baseline differences between groups as a standalone signaling question in its randomization domain (Domain 1). Instead, it focuses on evaluating key aspects of the randomization process, including sequence generation and allocation concealment, while also considering whether observed baseline differences may indicate underlying issues with randomization. This approach reflects the tool's emphasis on assessing the risk of bias arising from flaws in the randomization procedure rather than treating baseline imbalances as an independent source of bias. The rationale is that proper randomization should theoretically minimize systematic differences, so significant imbalances may raise concerns about the randomization process itself, but they are not automatically equated with bias.

**9. How did the authors treat the results of conditions for comparison (e.g., a result of the SE-VF group in Goo et al. (2024))? Were the results in those conditions excluded because more than one intervention was compared at the same time?**

**Response:** Thank you for your comment. Yes, based on Table 1 (PICOS criteria), the results of conditions with multiple simultaneous comparisons (e.g., the SE-VF group in Goo et al. (2024)) were excluded to maintain a clear comparison between single-intervention conditions.

**10. Were Kappa statistics conducted between two reviewers at any stage?**

**Response:** Yes, it was conducted for the inter rater between two reviewers when assessed risk of bias.

**11. Study identification and Figure 1: There is a discrepancy between the numbers reported in the text and those reported in Figure 1. Does each indicate something different?**

**Response:** Thank you for your insightful comment. We have made precise revisions to reflect the correct numbers.

“Based on the PRISMA guidelines [23], the electronic databases search process initially retrieved 791 articles, but after removing duplicates, 547 (72.6%) studies were left for further screening based on the inclusion and exclusion criteria.”

**12. Regarding the study referenced as number 30, which did not mention the sex of the participants, which sex group were that participants finally included into?**

**Response:** Thank you. The distribution of participants based on sex was revised. Here is the revised text:

“The nine studies included in this systematic review, published between 2018 and 2024, encompassed 367 participants. Among the 319 participants with reported sex distribution, 102 (32.0%) were male and 217 (68.0%) were female. Studies were conducted across Iran (n=4), Korea (n=2), Thailand (n=1), and India (n=2).”

**13. Table 4: There is a discrepancy between the certainty reported in the text (moderate) and those reported in Table 4 (Low) about JPS. Please correct the discrepancy.**

**Response:** Thank you for your comment. Here is the revised text:

“The certainty of the evidence was assessed using the GRADE approach. For the outcome of JPS, the certainty of evidence was rated as Low, due to some concerns about inconsistency related to variability in interventions and measurement tools. For JPE, the certainty of evidence was rated as low, primarily due to inconsistency and imprecision caused by small sample sizes and heterogeneous outcome measures. A summary of findings is presented in Table 5.”

**14. Key findings and mechanisms of improvement: This may be a typo: “ES= 1.12=3.66”.**

**Response:** Thank you for your comment. Here is the revised text:

“For example, Parishan et al. (2021) reported significant reductions in repositioning errors in flexion (ES= 1.12–3.66) after 4 weeks of stabilization training, suggesting that neuromuscular re-education plays a critical role in proprioceptive recovery”

**15. Limitations and future directions: I suppose that any assessment tools used by the included studies have a limitation that the current measurement tasks (i.e., Joint Position Error Test) cannot evaluate cervical proprioceptive acuity directly. Specifically, there is a possibility that the component of body sway may lead to measurement error, or the fact that it is difficult to isolate the influence of the vestibular system or motor control dysfunction. Are these inherent limitations of assessment tests not mentioned in the section on Limitations and future directions?**

**Response:** Thank you for highlighting this important point. We have now included a statement in the limitations section addressing that current proprioceptive assessments, like the Joint Position Error test, may be influenced by body sway, vestibular input, and motor control, which limits their ability to isolate cervical proprioception accurately. We have added this section to the limitation:

“Moreover, current assessment tasks such as the JPE test do not directly measure cervical proprioceptive acuity and may be influenced by confounding factors including body sway, vestibular input, and motor control dysfunction. These inherent limitations may introduce further measurement error and affect the accuracy of proprioceptive evaluation.”

**16. Figure 2:** **The black square and black triangle probably represent the mean or median.　Please specify what each symbol indicates and what the error bars demonstrate.**

**Response:** Thank you for your valuable comment. We have revised the figure to use the following symbols for clarity: ● represents each individual effect size, and — indicates the mean effect size. These updates are also reflected in the caption of Figure 2.

**17. References: The cited studies are sometimes misnumbered. Please check the reference list and corresponding numbers throughout the whole text.**

**Response:** Thank you for your comment. The reference list was checked and updated.

**#Reviewer 4**

**The manuscript is well structured , evidenced based , methodology aligns well with augment. I therefore endorse the manuscript for publication being very comprehensive structured to provide readers with a clear insight.**

**Response:** We sincerely appreciate your positive feedback and endorsement of our manuscript. It is gratifying to know that you found our work well-structured, evidence-based, and methodologically sound. Your recognition of the clarity and comprehensiveness of our systematic review is particularly encouraging, as we aimed to provide readers with a transparent and insightful synthesis of the current evidence on exercise interventions for cervical proprioception in individuals with forward head posture.
